# Let’s rise up to unite taxonomy and technology

**DOI:** 10.1371/journal.pbio.2002231

**Published:** 2017-08-18

**Authors:** Holly M. Bik

**Affiliations:** Department of Nematology, University of California, Riverside, Riverside, California, United States of America

## Abstract

What do you think of when you think of taxonomy? An 18th century gentlemen in breeches? Or perhaps botany drawings hung on the walls of a boutique hotel? Such old-fashioned conceptions to the contrary, taxonomy is alive today although constantly struggling for survival and recognition. The scientific community is losing valuable resources as taxonomy experts age and retire, and funding for morphological studies and species descriptions remains stagnant. At the same time, organismal knowledge (morphology, ecology, physiology) has never been more important: genomic studies are becoming more taxon focused, the scientific community is recognizing the limitations of traditional “model” organisms, and taxonomic expertise is desperately needed to fight against global biodiversity declines resulting from human impacts. There has never been a better time for a taxonomic renaissance.

From a historical perspective, the work of “traditional” taxonomists has focused on collecting, observing, and comparing species. Taxonomy extends far beyond simple drawings of body parts and encompasses the entire history of a species: reproductive strategies and larval development, feeding methods and ecological competition, as well as evolutionary musings on how and why a species came to be found in a particular place. Modern applications of taxonomy take many forms—microbiologists culturing new species, marine ecologists sorting specimens according to body plan, and phylum-specific experts searching the globe for their elusive group of organisms. Taxonomy is paradoxically cheap (requiring only a few buckets and a microscope) yet expensive (education and training for taxonomic experts is often measured in decades), and this type of quiet, observational science has molded many scientific arguments across the biological sciences.

Over the course of my research career, my encounters with formal taxonomy have been purely accidental. Unlike career taxonomists, I don’t have the patience for long hours at the microscope, and drawing the minutiae of morphological features (as is required for formal species descriptions) sends me into shudders. Yet, my PhD work on nematode worms and continuing collaborations with ecologists and morphological experts have turned me into an outspoken advocate of taxonomists and their creed. As I continue to delve deeper into the world of genomics and computational biology, I’ve had an epiphany: my research program cannot survive without taxonomy.

Taxonomic experts have invaluable specialist knowledge about specific groups of organisms. Their research focus may encompass one large and diverse phylum (think: nematodes or Platyhelminthes), span several smaller, obscure phyla (Kinorhyncha, Loricifera, Gnathostomulida), or be hyperfocused on a diverse class or subclass of organisms (polychaete worms, aplacophoran molluscs, copepod crustaceans). There are microbial taxonomists who study bacteria and single-cell protists, and the methods in which species are identified and described can vary substantially depending on the size of the organism.

Morphological taxonomy is critically important to many disciplines, so I am confident that it will survive in some form for years to come. Yet, all too often, I hear doom-and-gloom predictions about the slow death of taxonomy: its demise has been predicted for over a decade [1], and even optimistic reports about increasing numbers of taxonomists have been met with pleas of, “It still isn’t enough!” [2]. Yes, biodiversity loss and human impacts are accelerating faster than efforts to describe Earth’s species before they go extinct—and it is also true that the smaller the animal, the larger the taxonomic deficit. But those are not reasons to give up fighting. I have met many taxonomists who embrace the challenge and have become emboldened to merge their traditional expertise with cutting-edge techniques.

Why should we salvage a “dying” discipline? There are moral and practical reasons we should fight to preserve taxonomy.

First, and in the most practical sense, taxonomy *should* be revamped and reborn for the modern age. Morphological knowledge has limitations, and in some cases, it can be completely incorrect or misleading. Modern taxonomy must use DNA—molecular information is objective and easy to obtain, and it would be senseless to downplay the transformative contributions of nucleotide data. Depending on your budget, DNA-based information can be gathered quickly and cheaply (e.g., a US$6 Sanger sequence) or represent a deep and time-consuming project (>US$2,000 high-throughput Illumina dataset requiring substantial computational analysis). By integrating modern—Omics approaches with morphology-based knowledge, we can enhance and expand scientific insights gained from both disciplines. By forging strong links between morphological experts and computational genomics researchers, we can formulate broader, more globally relevant hypotheses. Interdisciplinary research efforts can (and should) accelerate the pace of traditional taxonomy, improve research efficiency, and—importantly for all scientists—lead to “fundable” cutting-edge research questions.

Second, by tapping the traditional taxonomic knowledge base, we will fundamentally improve database resources for all scientific disciplines, dramatically improving the accessibility of historical knowledge. Since Linnaeus founded taxonomy as we know it, there have been 280 years of human effort focused on morphological taxonomy: natural history drawings, identification keys, species descriptions, and monographs. Unfortunately, most of these important historical records are effectively invisible in the digital age. If it isn't online, it might as well not exist. This represents a stark limitation for molecular studies, which could reap massive benefits if such traditional, detailed knowledge from past taxonomic efforts could be incorporated into genomic data analysis. Modern—Omics studies would make maximal use of digitized, computationally accessible taxonomic metadata: geolocated species observations, records for type specimens, morphological characters and ecological traits, links to obscure photographs and drawings, and appropriate identification keys. For the moment, taxonomic knowledge remains haphazardly linked to—Omics datasets, mainly through (manual) Google searches and loose collaborations with experts. The digitization of taxonomic knowledge—made searchable and easily accessible via online portals and databases (Fig 1)—would serve to underline the value of taxonomy to a much broader swath of the scientific community.

**Fig 1:**
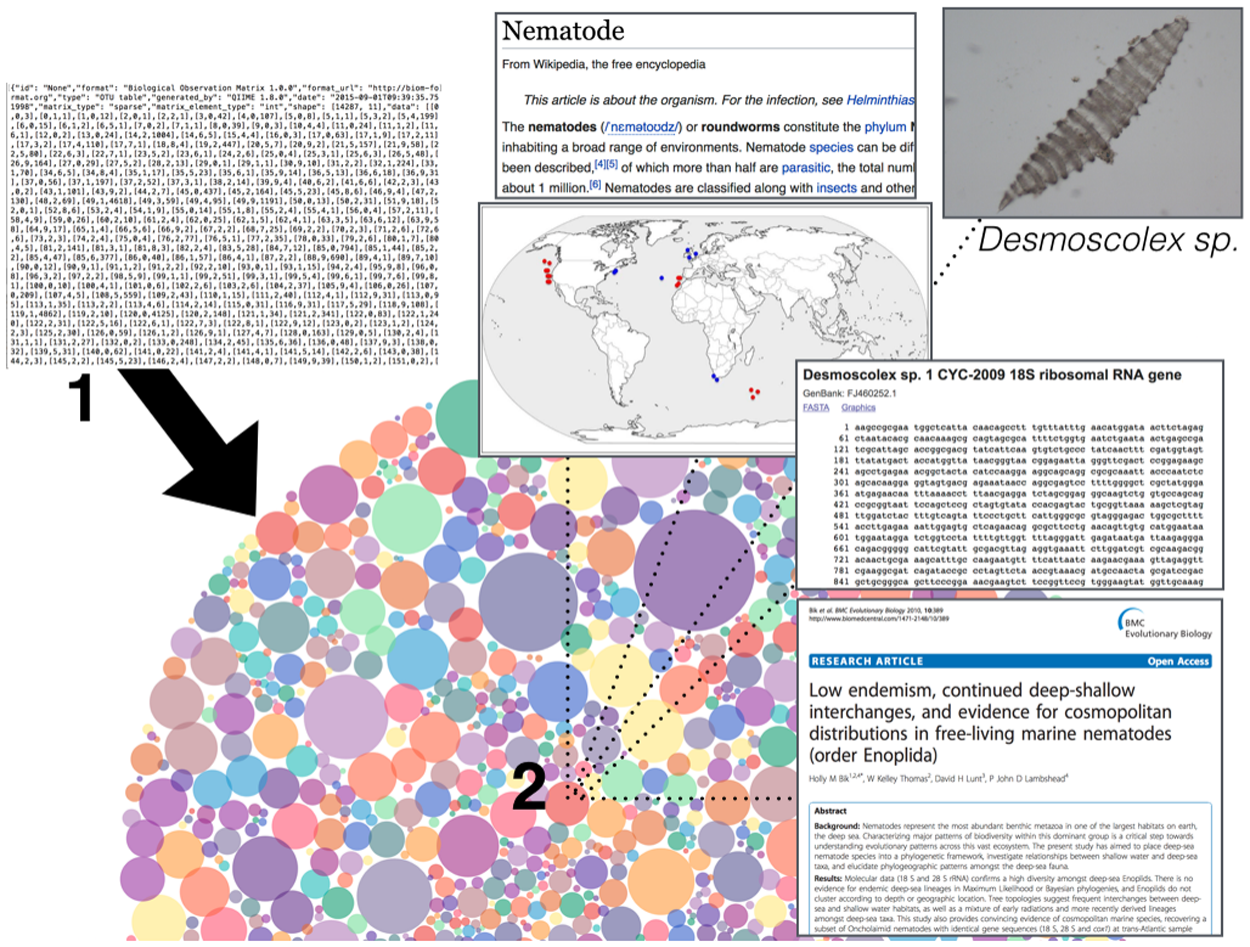
Schematic of data visualization portal that would integrate genomic information with digitized specimen records and morphological taxonomy. **(1)** Step 1: Text file outputs from—Omics bioinformatics pipelines are converted into a visual format. In this example, a tab-delimited text file containing operational taxonomic units (OTUs, which can be considered molecular “species”) is visualized as colored circles in a bubble chart in the Phinch framework [3], with circle size correlated to abundance. **(2)** Step 2: Clicking on a specific data point (e.g., an OTU) will pull up any online information associated with that species ID or taxonomic group, such as Wikipedia entries, photos, DNA sequences, peer-reviewed articles, and geolocated species observations displayed on a map. *Nematode image generated by Tiago Jose Pereira in the Bik Lab at UC Riverside; journal article screenshot and map with data points derived from* [4].

Third, technology has the power to bring 21st century taxonomy to the masses by merging and disseminating traditional knowledge alongside—Omics “big data.” Smartphones, tablets, and laptop computers are now ubiquitous—handheld computing power and intuitive user interfaces offer almost limitless potential for communicating datasets and scientific concepts. Digitized taxonomic resources (see above) could be made immediately accessible to nonspecialists (teachers, citizen scientists), making it easy to obtain previously obscure resources. In a similar vein, data-visualization tools (web portals, apps, etc.) represent a compelling but underdeveloped method for making “big data” accessible to broad audiences. Such visualization tools could empower noncomputational researchers (taxonomists, ecologists) and public audiences alike by presenting and summarizing information in novel ways (Fig 1). Technology could thus help to “rebrand” taxonomy for the modern age, reinventing taxonomy as an exciting, interdisciplinary research field rather than a cloistered discipline practiced by Victorian naturalists.

Finally, if we collectively shrug and (falsely) believe that taxonomy is in decline, then we are also inherently acknowledging the inferiority of basic science compared to more “applied” and “translational” disciplines. This is as much a moral argument as one with serious practical consequences. There is immense value in focusing basic research efforts on species-rich groups and microbial clades, because dramatic, transformative discoveries are most often accidental. Gene-editing tools facilitated by CRISPR [5], new classes of antibiotics, and new molecular biology tools for application in the biotech and bioengineering sectors (e.g., green fluorescent protein originally isolated from jellyfish [6]) have all resulted from basic “blue skies” research. Oftentimes a single species is the target of the investigation because researchers look into a unique or unusual feature of its biology. Taxonomy inherently emphasizes the diversity of form and function across the Tree of Life. It has taught us that experimental “model” organisms are not representative of the diversity within their phylum. For example, *Caenorhabditis elegans* is a very odd nematode indeed: a self-fertilizing hermaphrodite worm that can be easily grown on artificial media. In contrast, most nematode species are gonochoristic (individuals are either male or female), reproduce sexually, and do not survive under cultured lab conditions [7]. Traditional, morphological taxonomy celebrates rambling exploration and discovery. We must ensure that these fundamental components of science are not lost amidst the contemporary strains on scientific research.

Taxonomy could be on the brink of another golden age—if we play our cards right. As it is reinvented and reborn in the 21st century, taxonomy needs to retain its traditional organismal-focused approaches while simultaneously building bridges with phylogenetics, ecology, genomics, and the computational sciences. There is much to gain and little to lose by deeply integrating morphological taxonomy with high-throughput sequencing and computational workflows. Uniting taxonomy and technology will represent a dramatic advance for all research disciplines, improving the biological and ecological relevance of diverse study systems. Of course, this effort will be painstaking, frustrating, and difficult (not unlike formal species descriptions!), but the whole scientific community must nonetheless rise up to this effort. To embrace taxonomy—and its firm root in observational knowledge—is to preserve the very ideals of science itself.
